# A novel biomarker for pleural effusion diagnosis: Interleukin‐36γ in pleural fluid

**DOI:** 10.1002/jcla.24799

**Published:** 2022-12-07

**Authors:** Lun Guo, Qipan Zhang, Chengna Lv, Xudan Ma, Xuxiang Song, Jing Huang, Weili Chen, Chaofen Li, Qunli Ding

**Affiliations:** ^1^ School of Medicine Ningbo University Ningbo China; ^2^ Department of Pulmonary and Critical Care Medicine, The Affiliated Hospital of Medical School Ningbo University Ningbo China; ^3^ Department of Pharmacy, The Affiliated Hospital of Medical College Ningbo University Ningbo China; ^4^ Department of laboratory medicine Ningbo Ninth Hospital Ningbo China

**Keywords:** biomarker, cytokine, diagnosis, IL‐36γ, pleural effusion

## Abstract

**Background:**

Numerous studies have described the critical importance of interleukin (IL) ‐36γ in host defense against lung infections, but it is unknown whether it plays a role in infectious pleural effusion (IPE). This study aimed to examine the levels of IL‐36γ in pleural effusions of different etiologies and evaluate the diagnostic accuracy of IL‐36γ in the differential diagnosis of IPE.

**Methods:**

A total of 112 individuals was enrolled in this research. IL‐36γ levels in pleural fluids of all 112 patients were measured by enzyme‐linked immunosorbent assay (ELISA). We also characterized these markers' diagnostic values across various groups.

**Results:**

Patients with tuberculous pleural effusion (TPE) and parapneumonic effusion (PPE) had exhibited markedly higher IL‐36γ levels in their pleural fluid than the malignant pleural effusion (MPE) and transudative effusion patients. Furthermore, the IL‐36γ concentrations in TPE patients were evidently higher than in uncomplicated parapneumonic effusion (UPPE) patients but significantly lower than in complicated parapneumonic effusion (CPPE)/empyema patients. Pleural fluid IL‐36γ is a useful marker to differentiate TPE from UPPE, at a cut‐off value for 657.5 pg/ml (area under the curve = 0.904, *p* < 0.0001) with 70.0% sensitivity and 95.7% specificity.

**Conclusions:**

The elevated IL‐36γ in pleural effusion may be used as a novel biomarker for infectious pleural effusion diagnosis, particularly in patients with CPPE/empyema, and is a potentially promising biomarker to differentiate between TPE and UPPE.

## Graphical abstract

Interleukin (IL)‐36γ concentrations in pleural effusions from various etiologies detected by enzyme‐linked immunosorbent assay and probed the clinical value of pleural fluid IL‐36γ in the differential diagnosis of pleural effusion. The figure of the article was drawn by Figdraw (www.figdraw.com).

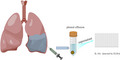

## 1 INTRODUCTION

Pleural effusions (PEs) can be caused by more than 60 diseases, making differential diagnosis difficult in the clinic.^1^ Using Light's criteria, transudative and exudative effusions may be distinguished.^2^ The etiology of exudative pleural effusions is more complex, which mainly includes parapneumonic effusion (PPE), tuberculous pleural effusion (TPE), and malignant pleural effusion (MPE). PPE is an exudative pleural effusion associated with an ipsilateral lung infection, which may be divided into three phases based on disease development and pathogenesis: uncomplicated parapneumonic effusion (UPPE), complicated parapneumonic effusion (CPPE), and thoracic empyema.^3^ It is essential to distinguish between UPPE and CPPE/empyema because it affects the choice of therapy decision; the former resolves with appropriate antibiotic therapy, while the latter two usually require pleural drainage or surgery.^4^ Clinically, it is hard to distinguish between TPE and UPPE since they have similarities in both clinical and laboratory terms, especially with negative cultures of *Mycobacterium tuberculosis* (*Mtb*) in PEs.

Interleukin (IL)‐36 cytokines include three pro‐inflammatory cytokines (IL‐36α, IL‐36β, and IL‐36γ) and one anti‐inflammatory cytokine (IL‐36 receptor antagonist, IL‐36Ra). The IL‐36 cytokines are generated at low levels in numerous organs and have variable expression patterns, with the highest expression and induction in the lung being IL‐36γ.^5^ Numerous studies have described the critical importance of IL‐36γ in host defense against lung infections caused by various pathogenic bacteria.^6^ IL‐36γ expression was discovered to be aberrant in numerous inflammatory and autoimmune disorders such as rheumatoid arthritis (RA),^7^ systemic lupus erythematosus (SLE),^8^ psoriasis,^9^ chronic obstructive pulmonary disease (COPD),^10^, ^11^ and acute respiratory distress syndrome (ARDS).^12^ Recent studies also found that IL‐36γ play a key role in type 2 diabetes mellitus (T2DM), which is related to chronic inflammation.^13^ In addition, IL‐36γ has been reported to promote macrophage killing of *Mtb* through non‐classical WNT signaling induced by WNT5A.^14^ Therefore, we selected IL‐36γ for this study to detect its levels in pleural effusions of different etiologies.

In the present study, the levels of IL‐36γ in PEs from various etiologies were measured, and identify this novel dysregulated cytokine serves as a potential biomarker for infectious pleural fluids, especially in patients who developed TPE and CPPE/empyema. Similarly, we found that IL‐36γ is a promising biomarker to distinguish TPE from UPPE.

## 2 MATERIALS AND METHODS

### 2.1 Participants

This prospective observational study was conducted from February 2019 to January 2022 at the Department of Respiratory and Critical Care Medicine of the Affiliated Hospital of Medical School, Ningbo University. The study initially enrolled patients who were undergoing sonography‐guided thoracentesis for PEs diagnosis. Patients without a definite diagnosis, those with hemothorax/chylothorax, and those with more than one possible etiology for the PEs are excluded. Ultimately, 112 individuals with PEs were enrolled in the research. The Ethical Committee of the Affiliated Hospital of Medical School of Ningbo University has authorized, and all patients' and/or their families' written consent were acquired before collecting the sample.

### 2.2 Etiological classification of pleural effusion

Patients were classified into five groups according to the cause of the pleural effusion: transudates (*n* = 20), MPE (*n* = 30), TPE (*n* = 30), UPPE (*n* = 23), CPPE/empyema (*n*  =  9).^1^ Transudate effusion was based on Light's criteria^2^ and the patient's general situation. The attending physician diagnosed transudate effusion and all of these patients were all secondary to cardiac failure in our investigation.^2^ MPE was identified as malignant cells, which were found in the pleural effusion or pleural biopsy specimen.^3^ TPE was diagnosed as an exudative pleural effusion along with the presence of a caseous granuloma in the pleural biopsy and/or a positive culture for *Mtb* in the pleural fluid or biopsy material or a positive sputum culture.^4^ PPE referred to exudative pleural effusion closely related to bacterial pneumonia, lung abscesses, and bronchiectasis and was further categorized as UPPE and CPPE/empyema by the pleural infectious stages. According to Light RW,^15^ CPPE/empyema was defined as the presence of PPE with one of the following additional criteria: (a) lactate dehydrogenase (LDH) > 1000 U/L; glucose <60 mg/dl; (b) pH<7.2; or (c) bacteria found on Gram's stain or culture. UPPE is considered to have found no organisms in a culture or on a Gram stain. In our study, infectious pleural effusion (IPE) was defined as PPE and TPE, whereas non‐infectious pleural effusion (NIPE) was defined as MPE and transudate.

### 2.3 Pleural fluid collection and Laboratory Examinations

Thoracentesis was employed to acquire pleural fluid samples from all patients before therapy. PE samples were promptly examined for the following biochemical indicators: pleural effusion total protein, LDH, adenosine deaminase (ADA), and glucose. Additional examinations were carried out, including a total and differential cell count as well as cytology and microbiologic research, including bacterial Gram's stain method and pleural fluid culture. The remaining pleural fluid was obtained and then centrifuged for 15 min at 1000*g*, after which the supernatants were aspirated and kept refrigerated at −80°C.

### 2.4 Measurements of IL‐36γ

IL‐36γ concentrations of PE were measured with enzyme‐linked immunosorbent assay (ELISA) kits (CUSABIO TECHNOLOGY LLC, Wuhan, China). In brief, each well was filled with 100 μl of a standard or a sample. At 37°C, the samples were then incubated for 2 h. Each well's liquid was removed, but not washed. Then each well was received 100 μl of Biotin‐antibody (1×). At 37°C, the plate was incubated for 1 h. Three times the plate was washed. Each well was received 100 μl of HRP‐avidin (1×). For 1 h, the plate was again incubated at 37°C. Five times the plate was washed. Each well was added 90 μl of TMB Substrate. For 15–30 min, the plates were then incubated at a temperature of 37°C (away from light). Each well was added 50 μl of Stop Solution.

An ELISA microplate reader (Thermo Fisher Scientific, Waltham, MA, USA) measured the OD value at 450 nm in 5 min for each well. Based on the standard curves, we determined the concentrations. The detected range for IL‐36γ was 78.0 pg/ml ‐ 5000.0 pg/ml, with a minimum detectable level of 19.5 pg/ml.

### 2.5 Statistical Analysis

Statistical analysis was analyzed by graphing software GraphPad Prism 8 (GraphPad Software Inc.). Categorical data were represented as frequency. The median (interquartile ranges) was used to represent quantitative data. The Mann–Whitney U test (comparison of two groups) and the one‐way ANOVA test (comparison of three or more groups) were used to compare quantitative data. The analysis of qualitative data using the chi‐square test was conducted. The Spearman's rank correlation coefficient analyzed the correlation between IL‐36γ and parameters in PE. We used receiver‐operating characteristic (ROC) curve analysis and calculated the area under the curve (AUC) to assess IL‐36γ ability to discriminate IPE from NIPE, TPE from UPPE, and CPPE/empyema from UPPE. Youden's index was used to identify critical biomarker levels. *p* < 0.05 was considered statistically significant.

## 3 RESULTS

### 3.1 Study participants' characteristics

A total of 112 participants with a single cause of PE was recruited for this research, including 32 PPE (23 UPPE, 9 CPPE/empyema), 30 TPE, 30 MPE, and 20 transudative effusions. The research participants' ages ranged from 19 to 94. The demographic data, clinical characteristics, and laboratory findings of the participants are shown in Table 1.

**TABLE 1:** Demographic and clinical characteristics

Variable	Transudate (*n* = 20)	MPE (*n* = 30)	TPE (*n* = 30)	PPE total (*n* = 32)	*p* Value	PPE (*n* = 32)		
						UPPE (*n* = 23)	CPPE/empyema (*n* = 9)	*p* Value
Age (years)	77 (68.2,88)	68.5 (61.7, 79)	42 (24.7, 65)	66 (55.5, 79)	<0.0001	65 (55, 79)	76 (57.5, 81)	0.4564
Gender (Male/female)	14/6	21/9	22/8	29/3	0.1770	22/1	7/2	0.1839
Pleural WBC (cells/μl)	620 (370, 1125)	750 (398, 1765)	1820 (1200, 2995)	4200 (1700,8900)	<0.0001	3630 (1565,8525)	6500 (27,400, 20,000)	0.0757
Pleural neutrophil (%)	8.0 (16.5,38.5)	16.0 (8.8, 29.8)	14.5 (8.8, 25.3)	65.0 (40.0, 83.0)	<0.0001	62.5 (27.8, 82.3)	80.0 (61.0, 85.0)	0.040
Pleural lymphocytes (%)	48.5 (29.3, 57.5)	39.0 (23.5, 57.0)	74.0 (68.0, 85.0)	15.0 (8.0, 40.0)	<0.0001	17.5 (9.5, 60.3)	13.0 (5.5, 26.0)	0.1630
Pleural monocytes (%)	16.5 (8.0, 40.0)	16.5 (8.0, 48.5)	5.0 (3.0, 8.6)	8.0 (3.0, 10.0)	<0.0001	8.5 (3.0, 10.0)	4.0 (1.5, 12.0)	0.9857
Pleural total protein (g/L)	20.7 (14.6, 25.1)	46.4 (34.6, 50.5)	50.6 (47.3, 54.4)	43.3 (30.1, 49.5)	<0.0001	43.3 (31.4, 49.7)	43.2 (25.3, 54.9)	0.8002
Pleural Glucose (mmol/L)	7.61 (6.70,9.24)	6.46 (5.75, 7.89)	4.89 (3.12, 5.90)	5.69 (1.34, 7.26)	0.0006	6.72 (5.08, 8.19)	0.97 (0.20, 2.22)	<0.0001
Pleural ADA (U/L)	4.5 (3.0, 5.0)	8.0 (5.8, 11.3)	44.5 (38.8, 53.3)	14.0 (10.0, 40.3)	<0.0001	14.0 (9.0, 18.0)	45.0 (36.0, 47.0)	<0.0001
Pleural LDH (U/L)	109 (87, 132)	252 (172, 441)	642 (377, 891)	732 (241, 1341)	<0.0001	431 (186, 893)	2449 (1316, 3054)	<0.0001
Pleural IL‐36γ (pg/ml)	121.9 (46.3, 196.5)	185.3 (155.9, 269.0)	818.5 (569.0, 1157.0)	368.0 (210.0, 1521.0)	<0.0001	336.0 (187.5, 569.0)	2959.0 (2902.0, 3494.0)	<0.0001

*Note*: Data are presented as median (interquartile range).

Abbreviations: ADA, adenosine deaminase; CPPE, complicated parapneumonic effusion; LDH, lactate dehydrogenase; MPE, malignant pleural effusion; PPE, parapneumonic effusion; TPE, tuberculous pleural effusion; UPPE, uncomplicated parapneumonic effusion; WBC, White blood cell count.

### 3.2 Concentrations of IL‐36γ cytokine in pleural fluid

**FIGURE 1:**
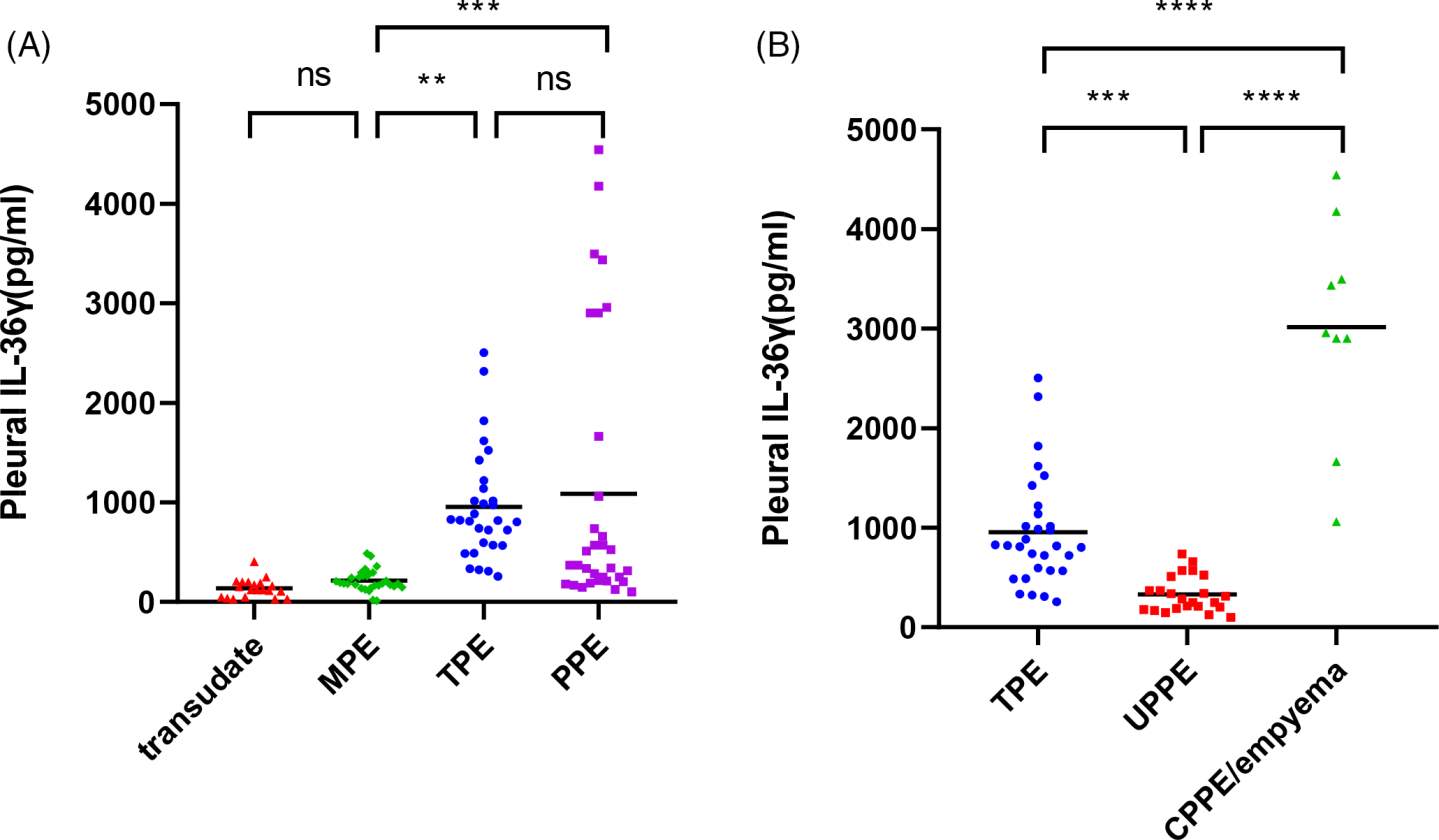
Pleural fluid IL‐36γ levels. (A) Pleural fluid IL‐36γ levels in patients with PPE, TPE, MPE, and transudate. (B) Pleural fluid IL‐36γ concentrations in patients with infectious effusions. Distribution (median, quartiles range). The plotting of individual values is conducted. The mean of the values is represented by the bars. ns, not significant. **p < 0.01, ***p < 0.001, ****p < 0.0001. CPPE, complicated parapneumonic effusion; MPE, malignant pleural effusion; PPE, parapneumonic effusion; TPE, tuberculous pleural effusion; UPPE, uncomplicated parapneumonic effusion.

The level of IL‐36γ in the pleural fluid was analyzed by sandwich ELISA. The pleural concentrations of IL‐36γ in individuals with PPE, TPE, MPE, and transudate were estimated as 368.0 (210.0, 1521.0) pg/ml, 818.5 (569.0, 1157.0) pg/ml, 185.3 (155.9, 269.0) pg/ml and 121.9 (46.3, 196.5) pg/ml, respectively (Table 1). The pleural level of IL‐36γ was significantly elevated in PPE and TPE compared with MPE and transudative effusion. However, there are no statistically significant differences were detected between the PPE group and the TPE group (*p* > 0.05) (Figure 1A). To further investigate the release of IL‐36γ in infected pleural effusions, we compared with plural fluid IL‐36γ levels in patients with TPE, UPPE, and CPPE/empyema (Figure 1B), the results revealed that the level of IL‐36γ in TPE group (*n* = 30) was statistically higher than those in UPPE group (*n* = 23), [818.5 (569.0, 1157.0) pg/ml vs. 336.0 (187.5, 569.0) pg/ml, *p* < 0.001], the level of IL‐36γ in CPPE/empyema was statistically higher than those in UPPE, [2959.0 (2902.0, 3494.0) pg/ml vs. 336.0 (187.5, 569.0) pg/ml, *p* < 0.0001]. However, it was similar between PPE and TPE groups, [368.0 (210.0, 1521.0) pg/ml vs. 818.5 (569.0, 1157.0) pg/ml, *p* > 0.05] (Table 1; Figure 1).

### 3.3 Correlation of IL‐36γ levels with biomarkers of pleural inflammation in IPEs

**FIGURE 2:**
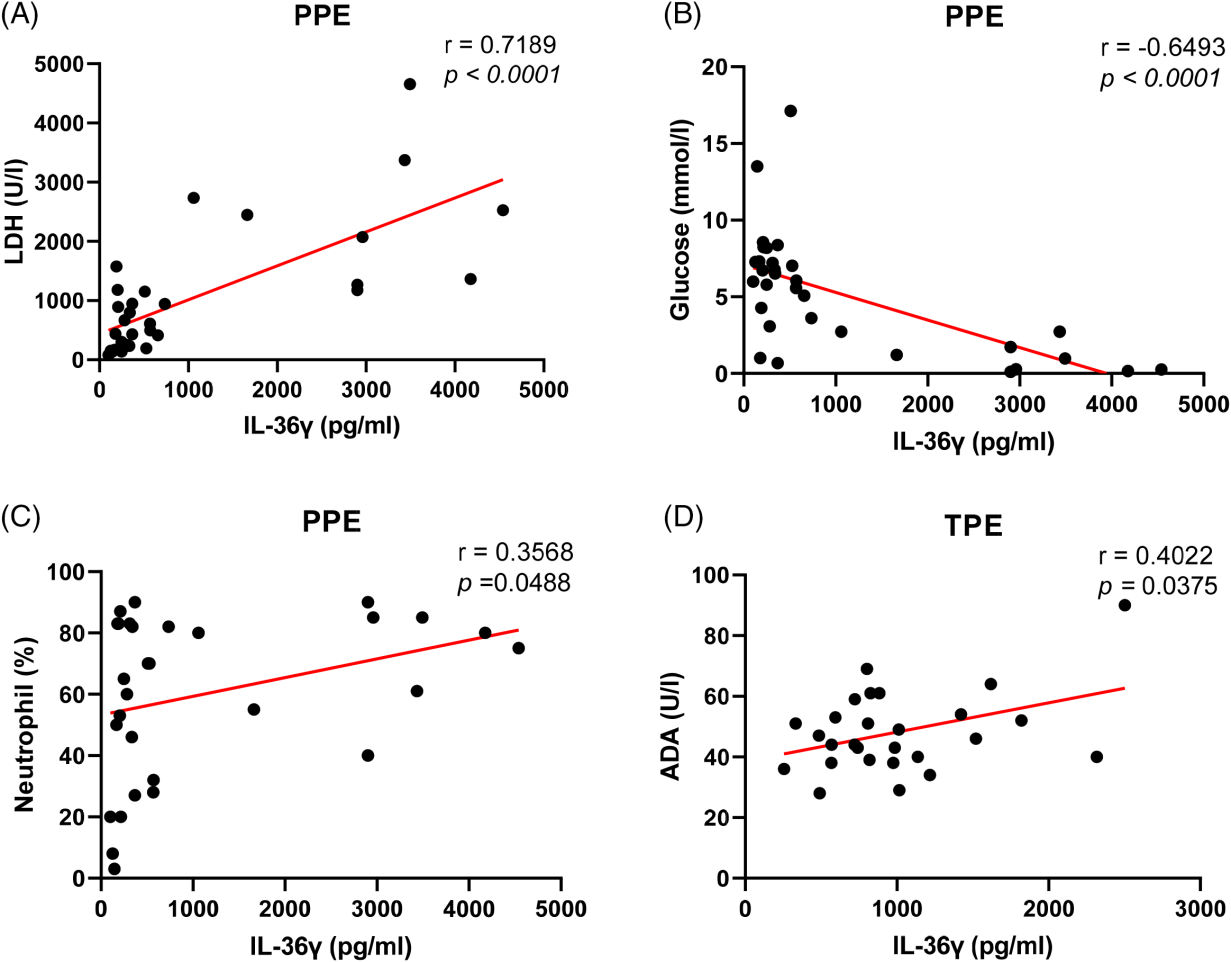
Correlation of IL‐36γ concentrations with markers of pleural inflammation in infectious patients.

Considering the increased IL‐36γ levels in all IPE patients (PPE and TPE groups), we also evaluated the relationship between pleural effusion IL‐36γ levels and pleural inflammation markers. Levels of IL‐36γ in pleural fluid were shown to be positively associated with LDH (*r* = 0.7189, *p* < 0.0001, Figure 2A) and inversely associated with glucose (*r* = −0.6493, *p* < 0.0001, Figure 2B) in PPE. In addition, we found a positive correlation between the pleural fluid IL‐36γ concentrations and the pleural fluid percentage of neutrophils in patients with PPE (*r* = 0.3568, *p* = 0.0488, Figure 2C). Pleural IL‐36γ positively correlates with pleural ADA in patients with TPE (*r* = 0.4022, *p* = 0.0375, Figure 2D).

### 3.4 Diagnostic value of IL‐36γ for pleural effusion differential diagnosis

**FIGURE 3:**
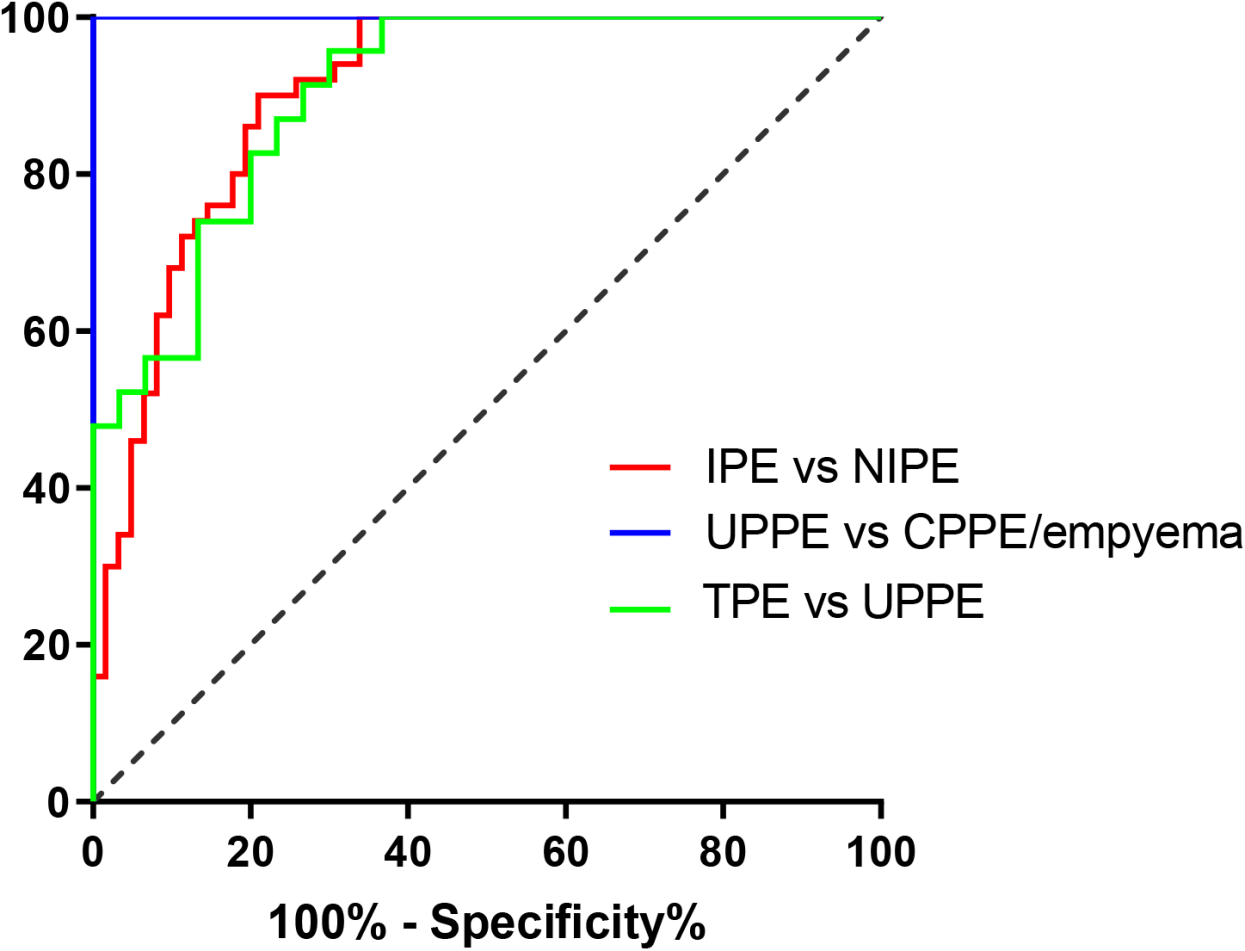
ROC curves of IL‐36γ concentrations for differential diagnosis of pleural effusion. NIPE, non‐infectious pleural effusion; IPE, infectious pleural effusion.

We assessed the effectiveness of pleural fluid IL‐36γ in differentiating IPE (PPE and TPE) from NIPE (MPE and transudate), TPE from UPPE, and UPPE from CPPE/empyema by using ROC curves (Figure 3), and the AUCs were 0.904 (*p =* 0.0278), 0.904 (*p* < 0.0001), and 1 (*p* = 0.0001), respectively. The optimal cut‐off values for determining IL‐36γ were 293.0 pg/ml for differentiating IPE and NIPE, 657.5 pg/ml for differentiating TPE and UPPE, and 736.0 pg/ml for differentiating UPPE and CPPE/empyema, respectively (Table 2).

**TABLE 2:** Diagnostic performance of IL‐36γ based on the ROC analysis

	Cut‐off value (pg/ml)	Sensitivity, % (95% CI)	Specificity, % (95% CI)	PPV (%)	NPV (%)	AUC	*p* value
IPE vs. NIPE	>293.0	79.0 (66.8–88.3)	90.0 (78.2–96.7)	90.7	77.6	0.904	0.0278
TPE vs. UPPE	>657.5	70.0 (50.6–85.3)	95.7 (78.1–99.9)	95.5	71.0	0.904	<0.0001
UPPE vs. CPPE/empyema	>736.0	100 (66.4–100)	100 (85.2–100)	100	100	1	0.0001

Abbreviations: AUC, the area under the curve; CI, confidence interval; IPE, infectious pleural effusion; NIPE, non‐infectious pleural effusion; NPV, negative predictive value; PPV, positive predictive value.

### 3.5 Diagnostic value of IL‐36γ, adenosine deaminase, and IL‐36γ combined with adenosine deaminase for Tuberculous Pleural Effusion

**FIGURE 4:**
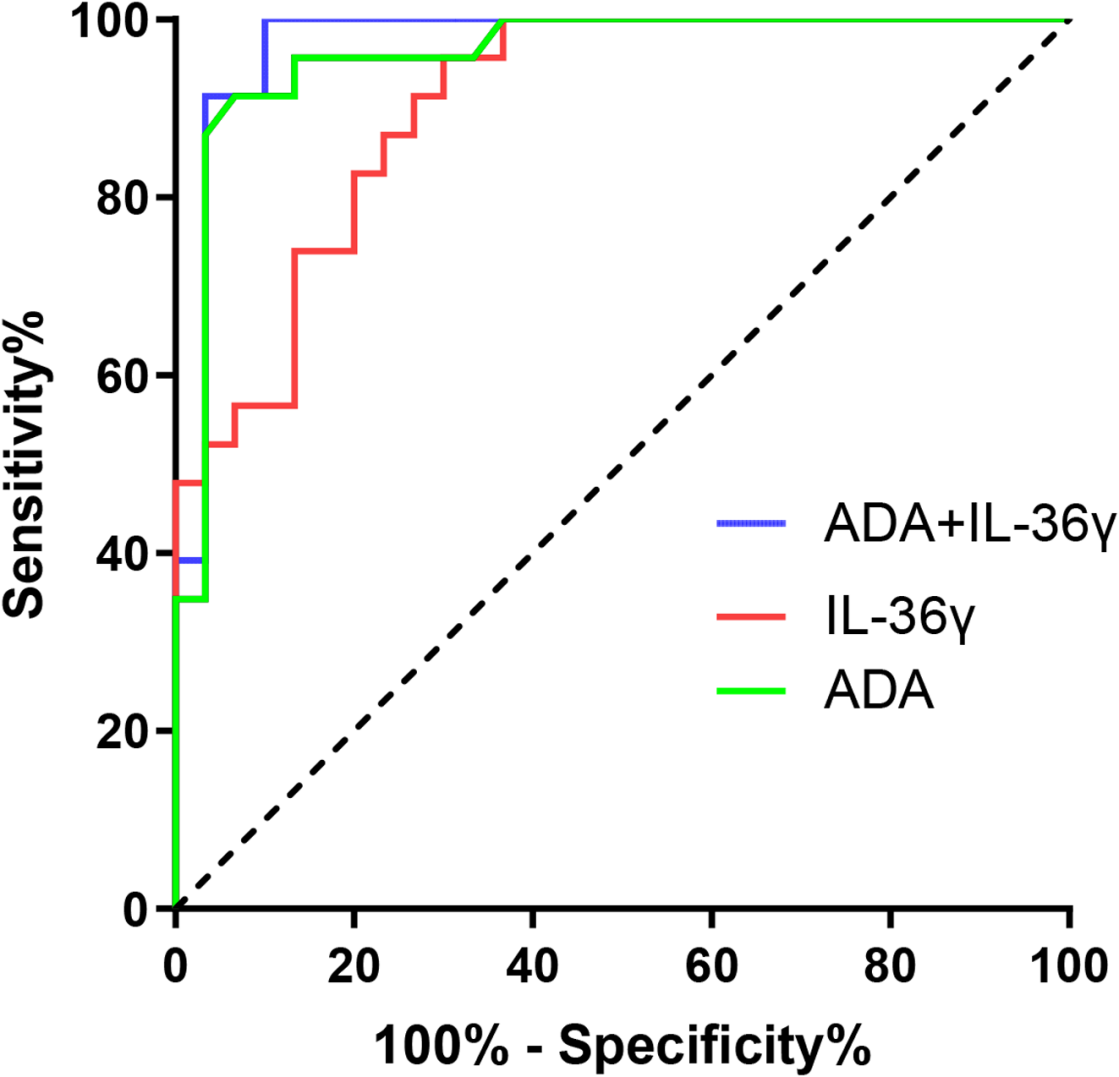
ROC curves of single and combined tests of pleural effusion IL‐36γ, ADA to diagnose TPE.

Since the ADA was the greatest individual predictor to diagnose TPE, we compared the diagnostic value of IL‐36γ versus ADA and examined whether the combination of the ADA and IL‐36γ might give even better prediction accuracy (Table 3). The ROC curve (Figure 4) showed that ADA combined with IL‐36γ (AUC = 0.974, *p* < 0.0001) divided TPE from the UPPE group better than either ADA (AUC = 0.959, *p* < 0.0001) or IL‐36γ (AUC = 0.904, *p* < 0.0001) alone. Furthermore, we determined by the optimal IL‐36γ cut‐off value in the pleural fluid at 657.5 pg/ml by ROC curve analysis. With this cut‐off value sensitivity of 70.0% and a specificity of 95.7% were obtained for TPE diagnosis compared with UPPE (Table 3).

**TABLE 3:** Diagnostic value of single and combined tests of pleural effusion IL‐36γ, ADA to distinguish from TPE and UPPE

Item	Cut‐off value	Sensitivity, % (95% CI)	Specificity, % (95% CI)	PPV (%)	NPV (%)	AUC	*p* Value
ADA	>28 U/L	93.3 (77.9–99.2)	91.3 (72.0–98.9)	93.3	91.3	0.959	<0.0001
IL‐36γ	>657.5 pg/ml	70.0 (50.6–85.3)	95.7 (78.1–99.9)	95.5	71.0	0.904	<0.0001
ADA + IL‐36γ	–	90.0 (73.5–97.9)	100 (73.5–97.9)	100	88.5	0.974	<0.0001

## 4 DISCUSSION

This is the first known study to detect the presence and different concentrations of IL‐36γ in pleural effusions. The concentration of pleural effusion IL‐36γ was increased in IPE (PPE and TPE). Among them, IL‐36γ concentration in the CPPE/empyema group was significantly higher than the UPPE group and might be used to differentiate CPPE/empyema from UPPE, meanwhile, the levels of pleural effusion IL‐36γ were shown to be substantially higher in TPE than UPPE. In addition, there was a good correlation between IL‐36γ pleural and inflammatory markers (LDH, glucose, and percentage of neutrophils) in patients with PPE, (*r* = 0.7189, *p* < 0.0001; *r* = −0.6493, *p* < 0.0001, and *r* = 0.3568, *p* = 0.0488, respectively). Also, pleural IL‐36γ was positively correlated with pleural ADA in patients with TPE, (*r* = 0.4022, *p* = 0.0375). Our data indicated that measurement of IL‐36γ in pleural effusions might help distinguish IPE from NIPE, TPE from UPPE, and CPPE/empyema from UPPE.

Exudative pleural effusions caused by inflammation of the lungs, such as bacterial pneumonia, lung abscesses and bronchiectasis infections, are known as PPEs.^16^ In addition, bacteria can invade the pleural cavity due to the increased permeability of the cell membrane, along with the increase of pleural LDH, pleural glucose, and pH drop, which can eventually lead to the formation of CPPE or even empyema.^17^ The inflammatory cytokine IL‐36γ has been linked to lung infections produced by a range of pathogenic microorganisms. Globally, *Streptococcus pneumoniae* is the most prevalent cause of community‐acquired pneumonia.^18^ During *Streptococcus pneumoniae* infection, IL‐36γ is rapidly produced in the lung by macrophages and is secreted into the extracellular space by packaging it in the form of particles and exosomes.^19^ IL‐36γ production in the lungs is also increased during *Klebsiella pneumoniae* lung infection.^20^ Our findings were that the pleural fluid IL‐36γ concentrations were significantly greater in CPPE/empyema than in UPPE. The cut‐off value of pleural IL‐36γ for the diagnosis of CPPE/empyema was 736.0 pg/ml with 100% sensitivity and 100% specificity. Our study shows that IL‐36γ can be used for early diagnosis of complicated pleural fluid and empyema, which can help us with early urokinase irrigation or adequate drainage as much as possible. Meanwhile, pleural fluid IL‐36γ concentration was positively correlated with LDH levels and neutrophil percentage, but adversely related to the pleural effusion of glucose levels. This raises the possibility that IL‐36γ is linked to the progression of PPE. Neutrophils are captured by activated endothelial cells and directed to the site of infection to form neutrophil extracellular traps (NETs).^21^ IL‐36γ can be processed and activated in NETs. It may have a significant impact on the development of PPE through self‐expression and activation, as well as the following coordination of the compliance signaling cascade.

TPE arises as a result of rupture of a subpleural parenchymal lesion that releases a small amount of *Mtb* entering the pleural cavity, triggering an immunological response on the local level. Neutrophil influx occurs firstly, Monocyte migration is followed by a significant T‐helper type 1 lymphocyte response.^22^ We found that IL‐36γ levels were substantially more in TPE groups than in UPPE groups, but lower than in CPPE/empyema. *Mtb* infection has been reported to trigger a Toll‐like receptor and myeloid differentiation primary response gene 88 (MyD88)‐dependent pathway in the host, leading to IL‐36γ secretion.^23^ We hypothesized that with the neutrophil influx in the early phase of TPE, IL‐36γ produced through the MyD88‐dependent pathway could promote its expression and activation, resulting in elevated IL‐36γ levels in TPE. Inflammatory pleural space lymphocytes concentrate and multiply, followed by a reduction in IL‐36γ levels as the TPE advances.

Our research still has many limitations. At the outset, the number of patients recruited for the study was limited. The sample numbers for the CPPE/empyema group, in particular, were insufficient to enable us to contrast them as independent groups to the MPE group and TPE group. Additionally, it was likewise a single cross‐sectional investigation. Ultimately, blood samples were not taken from any of the participants in our trial. There was no way to compare the IL‐36γ levels in the serum and the pleural fluid of patients who suffered from pleural effusions.

In summary, this is the first report to our knowledge of the diagnostical value of IL‐36γ in pleural effusion. We found higher levels of IL‐36γ in infected pleural fluid (PPE and TPE). We also discovered that IL‐36γ is involved with the inflammatory response in infected pleural fluid, implying that measurement of IL‐36γ may be helpful in early distinguishing CPPE/empyema from UPPE. Our data suggest that the combination of IL‐36γ and ADA is more valuable in the diagnosis of TPE compared to UPPE than either is alone. These results need to be validated by other independent studies, and further studies are necessary to investigate the underlying mechanisms of IL‐36γ in the development of pleural effusions.

## AUTHOR CONTRIBUTION

Lun Guo involved in data curation, formal analysis, and writing the original draft preparation. Qipan Zhang involved in methodology and validation. Chengna Lv involved in software and validation. Xudan Ma, Xuxiang Song, and Weili Chen involved in investigation. Jing Huang involved in conceptualization and visualization. Chaofen Li involved in resources and conceptualization. Qunli Ding involved in resources, supervision, conceptualization, reviewing, and editing.

## FUNDING INFORMATION

This work was supported by the Zhejiang Provincial Natural Science Foundation of China (No. LBY22H180004), Clinical Research and Application Project of Zhejiang Health Science and Technology Program (No. 2022KY1141) and Ningbo Natural Science Foundation (No. 2019A610237).

## CONFLICT OF INTEREST

None declared.

## Supporting information


Figure S1

## Data Availability

Data are available on request from the authors.
