# Supplementary figures and images for: A novel biomarker for pleural effusion diagnosis: Interleukin‐36γ in pleural fluid
Source: J Clin Lab Anal. 2022 Dec 7;37(1):e24799. doi: 10.1002/jcla.24799 (PMC9833963; doi:10.1002/jcla.24799)

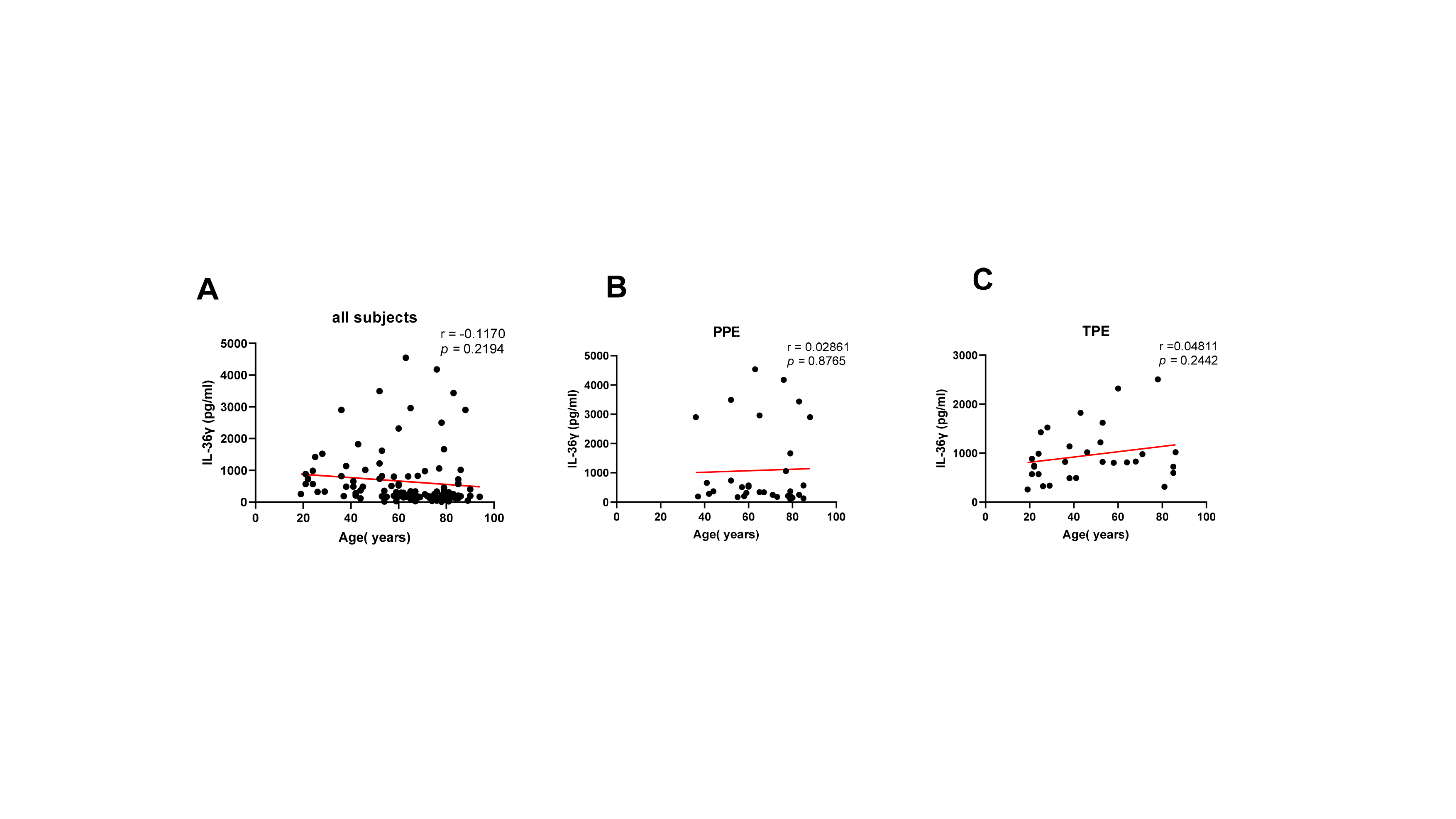

Supplement: Supplementary file 1 — Figure S1 [file JCLA-37-e24799-s001.tif]
